# Occurrence and spatial distribution of heavy metals in landfill leachates and impacted freshwater ecosystem: An environmental and human health threat

**DOI:** 10.1371/journal.pone.0263279

**Published:** 2022-02-03

**Authors:** Joseph P. Essien, Donald I. Ikpe, Edu D. Inam, Aniefiokmkpong O. Okon, Godwin A. Ebong, Nsikak U. Benson

**Affiliations:** 1 Department of Microbiology, University of Uyo, Uyo, Nigeria; 2 Department of Science Technology, Akwa Ibom State Polytechnic, Ikot Ekpene, Nigeria; 3 Department of Chemistry, University of Uyo, Uyo, Nigeria; 4 Department of Animal and Environmental Biology, University of Uyo, Uyo, Nigeria; 5 Department of Chemistry, Covenant University, Ota, Nigeria; Tsinghua University, CHINA

## Abstract

Municipal landfill leachates are a source of toxic heavy metals that have been shown to have a detrimental effect on human health and the environment. This study aimed to assess heavy metal contamination in leachates, surface water, and sediments from non-sanitary landfills in Uyo, Nigeria, and to identify potential health and environmental effects of leachate contamination. Over the wet and dry seasons, surface water and sediment samples were collected from an impacted freshwater ecosystem, and leachates samples from six monitoring wells. Elemental analyses of samples were conducted following standard analytical procedures and methods. The results indicated that leachate, surface water, and sediment samples all had elevated levels of heavy metals, implying a significant impact from landfills. Pollution indices such as the potential ecological risk index (PERI), pollution load index (PLI), degree of contamination (*Cd*), modified degree of contamination (*mCd*), enrichment factor (EF), geoaccumulation index (*Igeo*), and Nemerov pollution index (NPI) were used to assess the ecological impacts of landfill leachates. The following values were derived: PERI (29.09), PLI (1.96E-07), Cd (0.13), *mCd* (0.16), EF (0.97–1.79E-03), *Igeo* (0), and NPI (0.74). Pollution indicators suggested that the sediment samples were low to moderately polluted by chemical contaminants from the non-sanitary landfills, and may pose negative risks due to bioaccumulation. Human health risks were also assessed using standard risk models. For adults, children, and kids, the incremental lifetime cancer rate (ILCR) values were within the acceptable range of 1.00E-06–1.00E-04. The lifetime carcinogenicity risks associated with oral ingestion exposure to heavy metals were 9.09E-05, 1.21E-05, and 3.60 E-05 for kids, adults, and children, respectively. The mean cumulative risk values for dermal exposures were 3.24E-07, 1.89E-06, and 1.17E-05 for adults, children, and kids, respectively. These findings emphasized the risks of human and biota exposure to contaminants from landfills.

## 1. Introduction

Anthropogenic activities and processes tend to generate tons of waste that may consist of biodegradable and non-degradable to very hazardous wastes. However, the manner in which such wastes are collected, processed, stored, managed and disposed of, represents sources of potential environmental and human health risks. In metropolitan centers, particularly in rapidly expanding municipalities in developing countries, the dilemmas of solid waste management are of significant concern [1]. In most developing economies, it is a routine trend to dispose of household waste in poorly maintained and unregulated dumpsites, drainages, waterways, street piles, water channels, and concrete sidewalks [2]. However, in most developed nations, large-scale disposal of municipal solid wastes (MSW) may not pose a big problem due to the availability of waste management facilities. Given the likelihood that waste management facilities are sparse in developing countries, including Nigeria, the collection, processing and disposal of solid waste are mainly done through direct labour, intensive operations, and many opportunities for direct contact and exposure to hazardous substances are possible.

Non-sanitary landfilling, particularly open tipping dumpsites, is the most common method of waste management in developing countries such as Nigeria. There is widespread use of non-engineered and uncontrolled landfills without adequate bottom liners, leachate collecting, or treatment systems for proper management of municipal solid wastes. This has led to the generation of landfill leachates which have significant loads of organic and inorganic contaminants and are particularly hazardous to the environment [3,4]. Leachate is any substance that has seeped from decomposed wastes, dissolved or suspended materials. Landfill leachates are produced at dumpsites when the water mixes mostly with waste dumps [5,6]. The composition of leachates is variable but mostly comprises xenobiotic organic materials (e.g., halogenated organic compounds, PAHs, pharmaceuticals, plasticizers, etc.), highly infective microorganisms, emerging organic contaminants, heavy metal and other inorganic compounds [7–10]. In certain dumpsites, the leachate is comprised of fluid which has penetrated the open landfill from diverse external sources, including soil erosion, groundwater, precipitation, and wastewater generated from the organic waste decomposition [11,12]. Biodegradation of solid wastes produces landfill leachate, which can contaminate both surface water and underground water [3,13–15]. Potentially hazardous substances such as toxins, chemical pollutants, etiological products, endocrine disruptors, and emerging chemical contaminants associated with municipal wastewater, sewage and solid wastes may be spread in the environment from landfill leachates. Highly toxic plants, pests, bugs, rodents, microorganisms and endogenous pathogens are biohazards that may be found at open dumpsites [1,16].

Potentially toxic heavy metals are ubiquitous in the environment and have been widely reported in sediments, surface water and aquatic organisms [17–20]. However, heavy metals are frequently detected in municipal landfill leachates from hazardous waste landfills as well as solid waste dumpsites [21–24]. Toxic chemical compounds such as heavy metals and persistent organic compounds are released into the atmosphere and the environment when municipal solid wastes are burned or dumped in the open [2,25–28]. Leachates from municipal landfills have been reported to release toxic metals into the environment, posing serious threats to nearby lands and groundwater, and then to surface water [2]. Even though the impact of generated leachate is reduced to a certain degree from the source of its generation, it could still bear deleterious effects to the environment and the public health through accumulated toxicants to the underlying surface and groundwater contamination. Additionally, the presence of organic carbon waste could influence the taste and smell of underground water, while nitrogenous compounds could trigger eutrophication in surface waters [22,29].

These environmental endpoints are apparent in non-sanitary landfills that lack suitable lining materials, enabling raw toxic leachates to seep into the subsoil, underground water and contaminate them [23,30,31]. Once in the environment, heavy metals are bioaccumulative and could pose considerable risk to public health, e.g., acute toxicity, carcinogenicity and mutagenicity, detrimental effects to the growth of terrestrial and aquatic organisms [9,32–37]. Potentially toxic metals, such as arsenic, can lead to adverse health disorders, including heart disease, cancer as well as intestinal abnormalities, peripheral arterial disease, diabetes and high blood pressure [38–40].

Waste disposal and treatment are particularly acute in developing countries, owing to a lack of technological interventions and infrastructure. In Nigeria, the waste management system is rudimentary, with virtually no landfills equipped with good bottom liners. Many studies in Africa and the Middle East have documented the occurrence, ecotoxicological, and health risk assessment of heavy metals in soils, sediments, leachate wells, and surface waters impacted by non-sanitary municipal solid wastes landfills [2,25,41–45]. Thus, non-sanitary landfills could become a repository and point source of multiple types of chemical compounds, which could contaminate soil and groundwater through seepage of toxic leachates. Therefore, identifying, determining, and assessing the ecotoxicological profile of heavy metals around soils, surface- and groundwaters impacted by landfill leachates, and associated health risks posed by municipal dumpsites becomes significantly critical.

The primary objectives of this study were (i) to determine the concentrations of potentially toxic metals in landfill leachates, sediments, and surface water samples collected from an ecosystem that receives waste directly from municipal waste dumpsites, (ii) to assess the contamination levels of heavy metals using different pollution indices, (iii) to assess the ecotoxicological and human health risks associated with toxic metals in leachates and the receiving freshwater ecosystems using risk assessment models.

## 2. Materials and method

### 2.1 Samples collection

Leachates from six leachate wells located around MSW dumpsites were collected using a hand-held scoop. Each sample was transferred aseptically into clean sterilized containers (10-litre capacity), filtered to remove debris, marked, and stored at 4°C prior to analysis. For the stated purpose of this study, sediment and surface water samples were also collected to represent the identified impacted freshwater ecosystems using a Shipek grab sampler and sterile 1-litre plastic bottles, respectively. Each sampling location was labeled using Global Positioning System (GPS) Gramin e-Trex 10. Prior to transportation to the laboratory, the sediment samples were stored in glass containers and kept in coolers packed with ice blocks. The required standard quality control and quality assurance procedures were strictly followed during sample collection, preservation, and storage prior to extraction and analysis. Samples were collected during the dry season, and the elemental analysis was completed within 48 hours of the sample being collected.

### 2.2 Elemental analysis in leachates, water and sediment samples

Before the analysis, the sediment samples were oven-dried at 80°C for 48 hours in petri dishes and then carefully ground with a roller pin to disaggregate each sediment subsamples. The powdered samples were then passed through a 63 μm sieve. Each sediment subsample was digested using the wet digestion method as previously described [2,46,47], using a cocktail of HCl (6.0 mL), HNO_3_ (0.3 mL), and 20 mL of 5.0 M (1 M = 1 mol dm^-3^) HNO_3_ solutions. Total metal concentrations (As, Cd, Cr, Cu, Fe, Pb, Ni, and Zn) in the digested solutions were measured using the inductively coupled plasma atomic emission spectroscopy (ICP-AES). Experimental blanks were prepared and used to validate the sample preparation procedure. The calibration standards were prepared using serial dilutions of commercially available heavy metal stock solutions (1000 μg/mL BDH Grade). During the investigation, analytical grade chemicals from Sigma-Aldrich were used for extraction. Prewashing with distilled water and rinsing with 1% HNO_3_ (v/v) were performed on all glassware used in this study. Triplicate analyses were performed for all extracted samples to determine the accuracy/reproducibility of the metal digestion procedure, and the concentration of heavy metals in leachates, sediments and surface water samples were determined on a dry weight basis mg kg^-1^ and mgL^-1^. The detection limits for As, Cd, Cr, Cu, Fe, Pb, Ni, and Zn were 0.01, 0.05, 0.1, 0.01, 0.01 and 0.01 mgkg^-1^, respectively. The analytical recovery rates from spiked surrogates ranged between 93–97%.

### 2.3 Evaluation of toxic metal contamination using pollution indices

The assessment of the ecotoxicological contamination associated with heavy metals in landfill leachates, sediments and surface waters, potentially originating from the municipal solid waste dumpsites in the study area employed existing pollution risk indices including contamination factor [48], degree of contamination [17,18,48–51], Tomlinson’s pollution load index [52], geoaccumulation index [53], modified degree of contamination [54,55], enrichment factor [54], Nemerow pollution index [56], and potential ecological risk index [48,57–59]. The potential health risks to humans associated with daily exposure via dermal contact and oral ingestion routes were evaluated using the US Environmental Protection Agency’s model for risks assessment [60,61]. However, the target hazard quotient (THQ) was used to evaluate the noncancer risks posed by possible exposure to potentially harmful heavy metals via direct oral ingestion of leachates, sediments, and water by [60,62]. The model equations and specific gradations of the degree of contamination and associated ecotoxicological and human risks have been reported [2,58,63].

The contamination factor (CF) evaluates the contamination impact of a single metal in sediments. The index CF is a pollution measure defined as the ratio of the concentration of an individual heavy metal to its background concentration [48]. The CF is usually expressed as:

$$CF=\frac{C_{metal}}{C_{background}}$$

where *C*_*metal*_ is the metal concentration in the sediment and *C*_*background*_ represents the background concentration of the metal. A computed contamination factor higher than 6 indicates high sediment contamination, and a value between 3–6 expresses considerable sediment contamination, while CF values between 1–3, <1 indicate moderate and low sediment contaminations for the assessed element, respectively.

Tomlinson’s pollution load index (PLI) is an integrated index commonly used to assess the ecosystem’s quality in relation to the amount of anthropogenic heavy metal concentrations in sediments [52]. In general, the PLI is a standardized pollution indicator of a given sediment sample’s heavy metal ecotoxicity status, reflecting the concentration of an individual heavy metal will potentially exceed the average natural background concentration. The PLI, in general, can be used to analyze and assess the combined heavy metal contamination status of sediment samples collected. The PLI is mathematically expressed as the *n*^*th*^ root of the product of the *nCF*:

$$\sqrt[{n}]{{CF}_{1}\times {CF}_{2}\times {CF}_{3}\times {CF}_{4}\times \ldots \times {CF}_{n}}$$

where CF is the contamination factor and n is the number of heavy metals analyzed. The background concentration used in this study is the pre-anthropogenic concentration of heavy metals in shale sediment [64].

The geoaccumulation index (*Igeo*) is commonly applied for the assessment of sediment contamination by heavy metals [17,18,65–67]. The *Igeo* for respective individual heavy metal was calculated from the following equation:

$$I_{geo}={log}_{2}\left(\frac{C_{n}}{1.5B_{n}}\right)$$

where *C*_*n*_ is the concentration of n^th^ heavy metal in the sediment sample, 1.5 is a correction factor adopted to address possible variations in the background concentration of heavy metals attributed to lithogenic and anthropogenic effects, and *B*_*n*_ is the geochemical background concentration of the n^th^ heavy metal. The degree of sediment contamination is generally categorized into seven rankings according to the *Igeo* values, and extremely polluted (5 < *Igeo*), heavily to extremely polluted (4 < *Igeo* ≤ 5), heavily polluted (3 < *Igeo* ≤ 4), moderately to heavily polluted (2 < *Igeo* ≤ 3), moderately polluted (1 < *Igeo* ≤ 2), unpolluted to moderately polluted (0 < *Igeo* ≤ 1), and practically unpolluted (*Igeo* ≤ 0) [17,53,66,68].

The modified degree of contamination usually expressed as *mCd* symbolizes a standardized Håkanson [48] equation modified and proposed by [69] as a pollution index for evaluating the relative contamination levels at a specified sampling site [18]. Mathematically, *mCd* is written as:

$$mCd=\frac{\sum_{i=1}^{n}{CF}_{i}}{n}$$

For the assessment and quantification of modified degree of contamination in sediments, the following rankings have been developed: mCd ≥ 32 denotes an extremely high level of contamination; 16 ≤ mCd < 32 denotes an extremely high level of contamination; 8 ≤ mCd < 16 denotes an extremely high level of contamination; 4 ≤ mCd < 8 denotes a high level of contamination; 2 ≤ mCd < 4 denotes a moderate level of contamination; 1.5 ≤ mCd < 2 denotes a low level of contamination; and mCd < 1.5 denotes nil to very low levels of contamination.

The enrichment factor (EF) is often used to accurately evaluate the contributions of human activities to the heavy metal concentrations mostly in sediments of an aquatic ecosystem, and could be calculated using the mathematical expression:

$$EF=\frac{{\left(\frac{C_{metal}}{C_{Fe}}\right)}_{sample}}{{\left(\frac{C_{mbkg}}{C_{Febkg}}\right)}_{crust}}$$

where *C*_*metal*_ and *C*_*mbkg*_ are the heavy metal concentrations in the sediment sample and the background/baseline value, respectively. *C*_*Fe*_ and *C*_*Febkg*_ represents the Fe concentrations in the sample and the background/earth crust, respectively. For reference, the average crustal abundance values of heavy metals are usually used as background concentration of the elements. In this analysis, the average crustal abundance data reported by [64] was used as a background reference. The EF values were categorized as follows: <1 represents no enrichment, < 3 represents mild enrichment, 3–5 represents moderate enrichment, 5–10 represents moderately severe enrichment, 10–25 represents severe enrichment, 25–50 represents very severe enrichment, and >50 represents extreme enrichment, which may be due associated with anthropogenic activities.

### 2.4 Statistical analysis

The XLSTAT-Pro software AddinSoft, Inc., NY, USA, was used for statistical analysis. The interrelationships between heavy metals and sampling sites were investigated using the principal component analysis (PCA). The data for PCA were validated using the Kaiser–Meyer–Olkin (KMO) and Bartlett sphericity tests. A p <0.05 difference was considered significant. The observed dataset was also subjected to agglomerative hierarchical clustering (AHC) analysis using Ward’s method, with Euclidean distances (proximity matrix) as a measure of similarity between the heavy metals and sampling sites.

## 3. Results and discussion

### 3.1 Concentrations of heavy metals from waste leachate

The MSW leachate contained high concentrations of heavy metals. Fe with a mean concentration of 1473.16±413.59 mg/L was the most dominant contaminant, followed by Zn and Cd with mean contaminants loads of 23.88±14.40 and 7.12±4.8 mg/L, respectively. The least encountered heavy metal was As, with a mean concentration of 0.35±0.34 mg/L (Table 1). Potentially hazardous heavy metals are pervasive in landfill leachates from municipal landfills and solid waste dumpsites [21,24,70]. In a similar report, [23] showed that raw leachate from sanitary and non-sanitary landfills contained elevated amounts of As, Cr, Mn, Se, and Fe. In landfill leachate, essential elements including iron, zinc, chromium, copper, and manganese are often prevalent and biologically complexed and bioaccumulative, thus making them considerably bioavailable through the trophic food chains [71–73]. According to [71], elevated concentrations of toxic heavy metals including As, Cd, Hg, Ni, Se, and Pb were detected from monitoring leachate landfills with varying volumes of deposited wastes, with the substantial impact of leachates on groundwater attributed to increasing heavy metals. According to findings by [74], heavy metal concentrations in impacted ultisols from a municipal landfill were found to be higher than those in less impacted samples. Additionally [75], reported very high heavy metal contaminations associated with Fe, Mn, and Zn.

**Table 1:** Heavy metal concentrations (mg/L) in non-sanitary landfills leachate.

Metals	DLS-1	DLS-2	DLS-3	DLS-4	DLS-5	DLS-6	Mean±S.d
Pb	4.23	3.38	4.11	2.02	1.94	2.33	3.00±1.04
Cd	11.16	9.23	13.44	4.14	1.68	3.11	7.12±4.80
Ni	1.34	7.02	1.99	1.04	0.92	2.22	2.42±2.31
Cr	2.86	1.99	1.48	0.988	1.552	0.144	1.50±0.91
Zn	33.42	47.66	23.66	11.22	16.22	11.13	23.88±14.40
As	0.14	1.06	0.722	0.088	0.054	0.033	0.35±0.43
Fe	1644	2004	1822	1033	1022	1314	1473.16±413.59

Landfill leachate samples from Udo Street dumpsite (DLS-1, DLS-2, DLS-3).

Landfill leachate samples from Anua hospital dumpsite (DLS-4, DLS-5, DLS-6).

Furthermore, the high concentrations of heavy metals in non-sanitary landfill leachate, surface water, and impacted sediments may be due to human activities, including the use of agrochemicals, fertilizers, leaded fuel, and the industrial production of cement, steel, and chemicals. Other land-based sources of heavy metals include industrial emissions, surface runoffs, tyre wears, demolition wastes, discarded construction materials, and end-of-life electronic wastes [23,41,76–81].

### 3.2 Heavy metal concentrations in surface water

The results recorded revealed seasonal variations in the elemental concentrations of heavy metals between stations. Tables 2 and 3 show the distribution, mean, and standard deviation of heavy metals in MSW sediments impacted by leachates and wastewater during the dry and wet seasons, respectively. Fe, followed by Cr, had the highest mean concentrations of all metals analyzed in sediment samples collected during dry and wet seasons. The mean concentrations for Pb (1.89±0.79), Zn (0.22±0.39), Cr (4.19±3.37), Fe (10.77±4.47), Ni (0.63±0.45), As (0.03±0.01) and Cd (0.29±0.36) recorded in sediment samples collected during the dry season were slightly lower than Pb (2.58±1.23), Zn (0.44±0.59), Cr (4.82±3.72), Fe (11.78±5.37), Ni (0.73±0.51), As (0.04±0.01) and Cd (0.15±0.11) obtained during the wet season. However, with the exception of Fe, which was readily detected in surface water samples from MSW-impacted water bodies, and Pb, which was detected in water samples from Ibaoku stream, the majority of heavy metals detected in receiving water bodies were at trace levels or below detectable levels (Tables 4 and 5).

**Table 2:** Heavy metal concentrations (mg/kg) in sediment during the dry season.

**Metals**	**SDS-1**	**SDS-2**	**SDS-3**	**SDS-4**	**SDS-5**	**SDS-6**	**Mean**
Pb	0.98	3.02	2.44	2.22	1.48	1.22	1.89±0.79
Cd	1.02	0.09	0.14	0.31	0.16	0.03	0.29±0.36
Ni	0.84	1.06	0.59	1.12	0.07	0.12	0.63±0.45
Cr	6.99	9.41	4.22	1.99	1.53	1.02	4.19±3.37
Zn	0.08	0.08	1.03	0.08	0.03	0.04	0.22±0.39
As	0.03	0.03	0.05	0.03	ND	ND	0.03±0.01
Fe	16.66	15.46	10.44	7.44	9.44	5.23	10.77±4.47

Sediment samples from Ibaoku Stream (SDS 1, SDS 2, SDS 3).

Sediment samples from Anua Stream (SDS 4, SDS 5, SDS 6).

**Table 3:** Heavy metal levels (mg/kg) in sediment during the wet season.

Metals	SDS-1	SDS-2	SDS-3	SDS-4	SDS-5	SDS-6	Mean
Pb	1.34	3.22	4.56	2.74	1.29	2.34	2.58±1.23
Cd	0.34	0.09	0.16	0.25	0.06	0.05	0.15±0.11
Ni	1.05	1.22	0.98	1.02	0.08	0.07	0.73±0.51
Cr	7.86	9.89	6.38	2.38	1.44	0.98	4.82±3.72
Zn	0.17	1.06	1.33	0.06	0.008	0.012	0.44±0.59
As	0.02	0.06	0.04	0.05	ND	ND	0.04±0.01
Fe	17.25	14.66	15.57	11.11	9.68	2.45	11.78±5.37

**Table 4:** Heavy metal concentrations (mg/L) in landfill impacted surface water during the wet season.

Metals	SWS-1	SWS-2	SWS-3	SWS-4	SWS-5	SWS-6	Mean
Pb	0.24	0.03	0.02	<0.01	<0.01	<0.01	0.09±0.12
Cd	0.02	0.03	0.02	0.01	0.02	0.02	0.02±0.006
Ni	1.01	0.04	0.04	<0.01	<0.01	<0.01	0.36±0.56
Cr	0.08	0.11	0.09	<0.01	0.03	<0.01	0.07±0.03
Zn	0.025	0.013	0.022	0.04	0.05	0.02	0.02±0.01
As	<0.01	<0.01	<0.01	<0.01	<0.01	<0.01	ND
Fe	1.55	0.88	1.44	0.062	0.032	0.015	0.66±0.72

**Table 5:** Heavy metal concentrations (mg/L) in landfill impacted surface water during the dry season.

Metals	SWS-1	SWS-2	SWS-3	SWS-4	SWS-5	SWS-6	Mean
Pb	0.09	0.43	<0.01	<0.01	<0.01	<0.01	0.26±0.24
Cd	0.03	ND	0.01	<0.01	<0.01	<0.01	0.02±0.01
Ni	<0.01	<0.01	<0.01	<0.01	<0.01	<0.01	ND
Cr	0.03	0.23	0.04	<0.01	<0.01	<0.01	0.1±0.11
Zn	0.01	<0.01	0.01	<0.01	0.02	<0.01	0.01±0.005
As	<0.01	<0.01	ND	<0.01	<0.01	<0.01	ND
Fe	2.12	1.03	0.89	0.02	0.01	0.01	0.68±0.84

### 3.3 Ecotoxicological status of heavy metals in landfill impacted freshwater sediment

Health risks associated with carcinogenic and noncarcinogenic contaminants were evaluated using EPA-developed standard risk models [62]. As indicated in Table 6, carcinogenic risks were estimated for oral ingestion (EDD_*ing*_) and dermal contact (EDD_*dermal*_) exposures. Noncarcinogenic risk assessment, on the other hand, is typically expressed in terms of the ratio of the determined dose of a contaminant to the reference dose (*R*_*f*_*D*) below which they are unlikely to pose any significant health risk. The present study adopted the target hazard quotient (THQ) method for assessing noncancer risk associated with determined toxic metals. The values presented in Table 6 revealed lower risk when compared with the impacted soil indices. The results recorded for adults, children and kids were within the acceptable incremental lifetime cancer rate (ILCR) range of 1.00×10^−6^–1.00×10^−4^. The carcinogenic lifetime risks among kids via oral ingestion recorded mean cumulative risk value of 9.09E-05 while values of 3.60E-05 and 1.21E-05 were obtained for children and adults. For dermal exposures, cumulative risk average values of 3.24E-07, 1.89E-06 and 1.17E-05 were recorded for adults, children and kids, respectively.

**Table 6:** Elemental carcinogenic lifetime risk and cumulative risk for different exposure pathways in kids, children and adults to MSW dumpsite sediment.

Kids (<1–6 years)							Children (<7–18 years)				Adults			
			Oral Ingestion		Dermal Exposure		Oral Ingestion		Dermal Exposure		Oral Ingestion		Dermal Exposure	
Metal	Conc. levels	Conc. (mg/kg)	EDD_ing_ (mg/kg-day)	THQ	EDD_dermal_ (mg/m^3^-day)	THQ	EDD_ing_ (mg/kg-day)	THQ	EDD_dermal_ (mg/m^3^-day)	THQ	EDD_ing_ (mg/kg-day)	THQ	EDD_dermal_ (mg/m^3^-day)	THQ
Pb	Min.	0.98	4.95 E-06	1.41 E-03	1.66 E-07	3.16 E-04	1.96 E-06	5.59 E-04	2.67 E-08	5.10 E-05	6.71 E-07	1.92 E-04	4.59 E-09	8.75 E-06
	Max.	3.02	1.52 E-05	4.35 E-03	5.12 E-07	9.75 E-04	6.03 E-06	1.72 E-03	8.25 E-08	1.57 E-04	2.06 E-06	5.91 E-04	1.41 E-08	2.69 E-05
	Mean	1.89	9.54 E-06	2.72 E-03	3.20 E-07	6.10 E-04	3.77 E-06	1.08 E-03	5.16 E-08	9.83 E-05	1.29 E-06	3.69 E-04	8.85 E-09	1.68 E-05
Cd	Min.	0.03	1.51 E-07	1.51 E-03	8.48 E-10	8.48 E-05	5.99 E-08	5.99 E-05	1.37 E-10	1.37 E-05	2.05 E-08	2.05 E-05	2.34 E-11	2.34 E-06
	Max.	1.02	5.15 E-06	5.13 E-03	2.88 E-08	2.88 E-03	2.04 E-06	2.04 E-03	4.65 E-09	4.65 E-04	6.98 E-07	6.98 E-04	7.96 E-10	7.96 E-05
	Mean	0.29	1.46 E-06	1.46 E-03	8.19 E-09	8.19 E-04	5.79 E-07	5.79 E-04	1.32 E-09	1.32 E-04	1.98 E-07	1.98 E-04	2.26 E-10	2.26 E-05
Ni	Min.	0.07	3.53 E-07	1.77 E-05	6.92 E-07	1.28 E-04	1.39 E-07	6.99 E-06	1.12 E-07	2.06 E-05	4.79 E-08	2.39 E-06	1.91 E-08	3.54 E-06
	Max.	1.12	5.65 E-06	2.83 E-04	1.11 E-05	2.05 E-03	2.24 E-06	1.12 E-04	1.78 E-06	3.31 E-04	7.67 E-07	3.83 E-05	3.06 E-07	5.67 E-05
	Mean	0.63	3.17 E-06	1.59 E-04	6.23 E-06	1.15 E-03	1.26 E-06	6.29 E-05	1.00 E-06	1.86 E-04	4.32 E-07	2.16 E-05	1.72 E-07	3.18 E-05
Cr	Min.	1.02	5.15 E-06	1.71 E-03	1.15 E-06	1.92 E-02	2.04 E-06	6.79 E-04	1.86 E-07	3.09 E-03	6.98 E-07	2.33 E-04	3.18 E-08	5.31 E-04
	Max.	9.41	4.74 E-05	1.58 E-02	1.06 E-05	1.77 E-01	1.88 E-05	6.27 E-03	1.71 E-06	2.86 E-02	6.44 E-06	2.15 E-03	2.94 E-07	4.89 E-03
	Mean	4.19	2.11 E-05	7.05 E-03	4.73 E-06	7.89 E-02	8.37 E-06	2.79 E-03	7.63 E-07	1.27 E-02	2.87 E-06	9.57 E-04	1.31 E-07	2.18 E-03
Zn	Min.	0.03	1.51 E-07	5.04 E-07	1.69 E-08	2.83 E-07	5.99 E-08	1.99 E-07	2.73 E-09	4.55 E-08	2.05 E-08	6.85 E-08	4.68 E-10	7.81 E-09
	Max.	1.03	5.19 E-06	1.73 E-05	5.82 E-07	9.70 E-06	2.06 E-06	6.86 E-06	9.38 E-08	1.56 E-06	7.05 E-07	2.35 E-06	1.61 E-08	2.68 E-07
	Mean	0.22	1.11 E-06	3.70 E-06	1.24 E-07	2.07 E-06	4.39 E-07	1.46 E-06	2.00 E-08	3.34 E-07	1.51 E-07	5.02 E-07	3.43 E-09	5.73 E-08
As	Min.	0.03	1.51 E-07	5.05 E-04	2.54 E-08	2.07 E-04	5.99 E-08	1.99 E-04	4.09 E-09	3.33 E-05	2.05 E-08	6.85 E-05	7.03 E-10	5.71 E-06
	Max.	0.05	2.52 E-07	8.41 E-04	4.24 E-08	3.45 E-04	9.98 E-08	3.33 E-04	6.83 E-09	5.55 E-05	3.42 E-08	1.14 E-04	1.17 E-09	9.52 E-06
	Mean	0.02	1.01 E-07	3.36 E-04	1.69 E-08	1.38 E-04	3.99 E-08	1.33 E-04	2.73 E-09	2.22 E-05	1.37 E-08	4.56 E-05	4.68 E-10	3.80 E-06
Fe	Min.	5.23	2.64 E-05	3.77 E-05	1.48 E-07	1.48 E-04	1.04 E-05	1.49 E-05	2.38 E-08	2.38 E-05	3.58 E-06	5.11 E-06	4.08 E-09	4.08 E-06
	Max.	16.66	8.41 E-05	1.20 E-04	4.71 E-07	4.71 E-04	3.33 E-05	4.75 E-05	7.59 E-08	7.59 E-05	1.14 E-05	1.63 E-05	1.30 E-08	1.30 E-05
	Mean	10.78	5.44 E-05	7.77 E-05	3.05 E-07	3.05 E-04	2.15 E-05	3.07 E-05	4.91 E-08	4.91 E-05	7.38 E-06	1.05 E-05	8.42 E-09	8.42 E-06
Cumulative risk for min. values			3.73 E-05		2.20 E-06		1.48 E-05		3.55 E-07		5.06 E-06		6.09 E-08	
Cumulative risk for max. values			1.63 E-04		2.34 E-05		6.45 E-05		3.76 E-06		2.21 E-05		6.45 E-07	
Cumulative risk for mean values			9.09 E-05		1.17 E-05		3.60 E-05		1.89 E-06		1.21 E-05		3.24 E-07	
												
HI min. value			3.84 E-03		2.01 E-02		1.52 E-03		3.24 E-03		5.21 E-04		5.56 E-04	
HI max. value			2.66 E-02		1.84 E-01		1.05 E-02		2.97 E-02		3.61 E-03		5.08 E-03	
HI mean. Value			1.18 E-02		8.19 E-02		4.68 E-03		1.32 E-02		1.60 E-03		2.27 E-03	

EDD_*ing*_−Ingestion estimated daily dose; EDD_*dermal*_−Dermal contact estimated daily dose; THQ–Target hazard quotients; HI–Hazard index.

**Fig 1:**
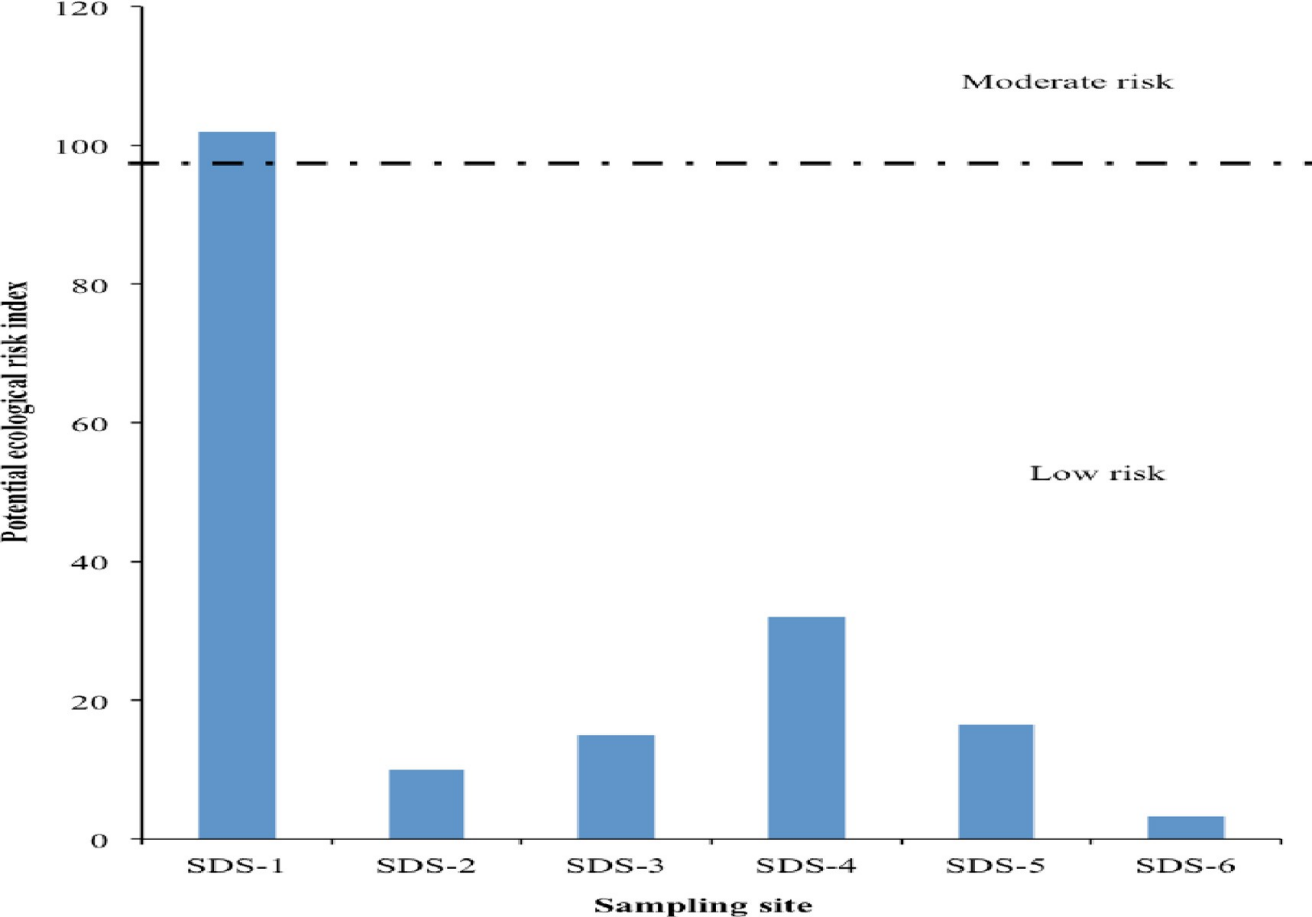
Ecological risk index (RI) of heavy metals in MSW dumpsite impacted sediment.

**Fig 2:**
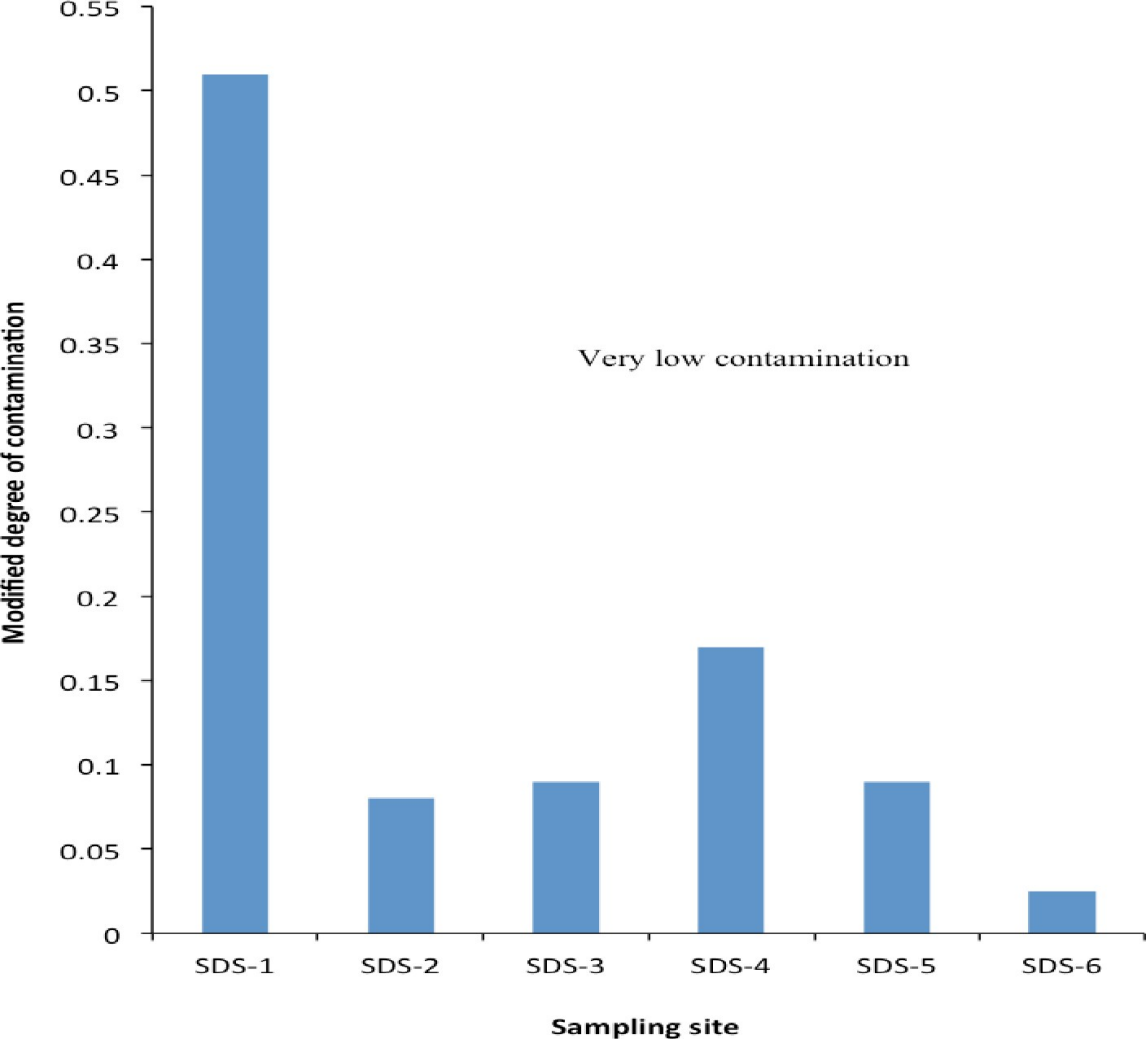
Modified degree of contamination of heavy metals in MSW dumpsite impacted sediment.

Table 7 presents the pollution indicators in sediment of the freshwater stream with a pollution load index, degree of contamination, Nemerov pollution index, and potential ecological risk index recorded as 1.96E-07, 1.13, 0.74, and 29.80, respectively. These results indicate that sediment samples from the Ibaoku stream location SDS-1 are laden with harmful heavy metals load and may pose considerably high risks to public health and the environment (Fig 1). The calculated modified degree of contamination (*mCd*) of 0.16 also revealed a possible moderate risk to public health (Fig 2). The Nemerov pollution index of 0.74 was very low while the *mCd* of 1.16 revealed moderate risks for sediment samples collected from location SDS-1. The mean enrichment factors (EF) of As, Cd, Cr, Ni, Pb, and Zn with reference to background concentrations in shale sediment showed poor enrichment as all the metals determined had EF values less than 1.5, indicating that they are more of crustal origin (Table 7). The calculated geoaccumulation index (*Igeo*) recorded relatively low values (<0) for most elements except Cd (>1.0) in sediment sample from location SDS-1 (Table 8), indicating that the sediments of the freshwater ecosystem within the MSW impacted environ might be moderately contaminated due to anthropogenic activities.

**Table 7:** Pollution indicators of heavy metals contamination of MSW dumpsite impacted sediment.

		Pb	Cd	Ni	Cr	Zn	As	Fe
Contamination factor		9.46 E-02	9.72 E-01	9.31 E-03	4.66 E-02	2.35 E-03	1.79 E-03	2.28 E-04
Degree of contamination	1.13							
Modified degree of Contamination	0.16							
Pollution load index	1.96 E-07							
Ecological risk factor		0.47	29.16	0.05	0.09	0.002	0.02	-
Potential ecological risk index (DSS1 –DSS9)	29.80							
Enrichment factor		0.09	0.97	9.31 E-03	4.65 E-02	2.35 E-03	1.79 E-03	-
Nemerov pollution index (DSS1 –DSS9)	0.74							

**Table 8:** Geoaccumulation indices (*Igeo*) of heavy metals in MSW sediment at investigated sites.

	SDS-1	SDS-2	SDS-3	SDS-4	SDS-5	SDS-6
Pb	-4.93	-3.31	-3.62	-3.76	-4.34	-4.62
Cd	1.18	-2.32	-1.68	-0.54	-1.49	-3.91
Ni	-6.92	-6.59	-7.43	-6.50	-10.51	-9.73
Cr	-4.27	-3.84	-4.99	-6.08	-6.46	-7.05
Zn	-10.80	-10.79	-7.11	-10.79	-12.21	-11.79
As	-9.34	-9.34	-8.60	-9.34	0	0
Fe	-12.05	-12.16	-12.72	-13.22	-12.87	-13.72

**Fig 3:**
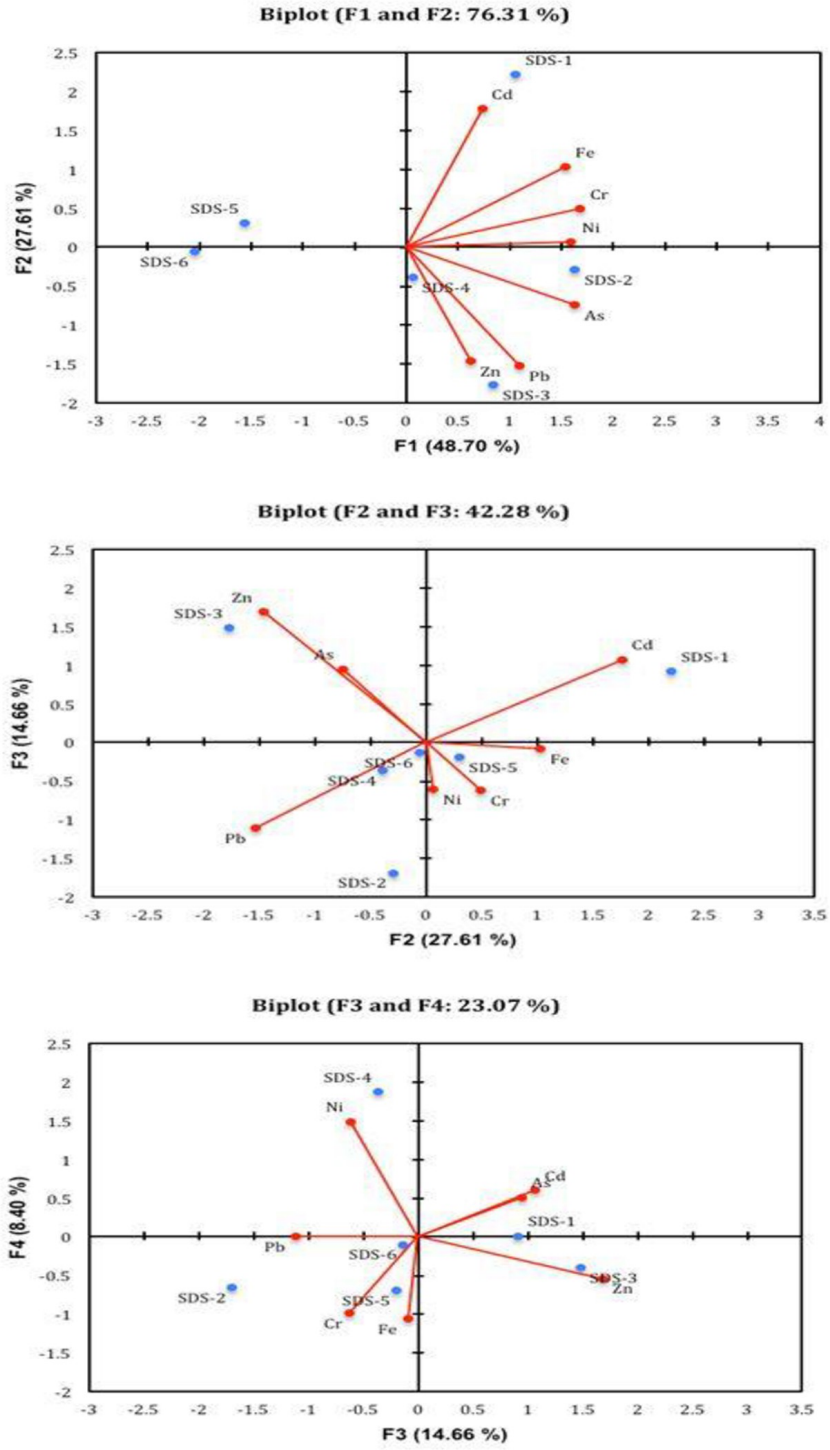
The two principal components reflecting the relationship of study sites and heavy metals (variables) in sediment samples.

Fig 3 presents the computed principal components describing the relationships between heavy metals (variables) and the sampling points. The first principal component accounted for 48.70 percent of the total variance and was positively correlated with Cd, Cr, Fe, and Ni loadings.

**Fig 4:**
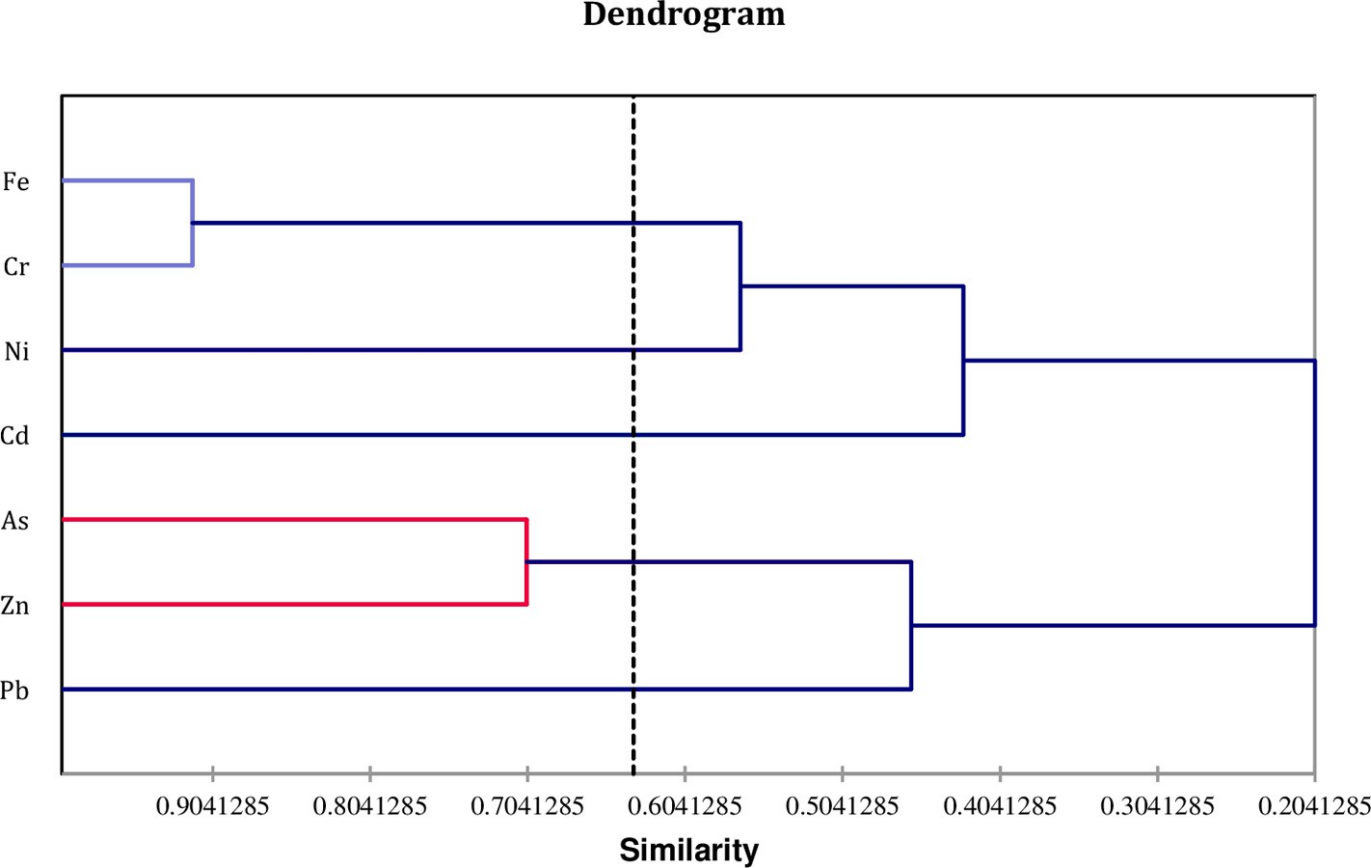
Hierarchical clustering analysis (Ward’s Method) showing the relevant association among heavy metals of sediment samples. (Distance metrics are based on the Euclidean distance single linkage method (proximity matrix).

This indicates that the heavy metals contamination at the SDS-1—SDS-3 landfill sites originated predominantly from anthropogenic sources to the leachate impacted sediments of the investigated ecosystems. The second principal component, which accounted for 27.61 percent of the total variance, exhibited a negative relationship between heavy metal contamination and sediment samples from the SDS5—SDS-6 sites. The present study revealed no significant differences in heavy metal concentrations in sediment samples across the landfill leachate impacted sites. However, the Ward method’s hierarchical clustering analysis revealed a significant and site-specific link between heavy metals in the analyzed sediment samples. The results revealed Fe, Cr and Cd as dominant sedimentary heavy metals (Fig 4).

### 3.4 Ecotoxicity status of heavy metals in the surface water and MSW leachate

Analysis has shown that the concentrations of heavy metals in surface water and MSW leachate samples may be toxic to humans. Hazard quotients of their toxicity to adults and children are given in Table 9. The probable hazard index established for adults and children who may be exposed to contaminated water during the dry and wet seasons, as well as exposure to MSW leachates, is relatively significant and may pose serious long-term health effects (Table 9).

**Table 9:** Hazard quotient associated with heavy metal exposure in adults and children.

		Water (wet)	Water (dry)	Water (dry 2)	Leachate
**ADULTS**	Pb	4.23E-02	7.41E-02	1.50E+00	2.38E+00
	Cd	1.14E+00	8.00E-01	1.67E+01	4.07E+02
	Ni	2.67E-01	1.43E-02	9.05E-01	3.46E+00
	Cr	1.05E+00	1.05E+00	7.99E+01	2.86E+01
	Zn	2.70E-03	1.11E-03	2.13E-02	2.27E+00
	As	9.52E-01	9.52E-01	3.33E+00	3.33E+01
	Fe	2.71E-02	2.78E-02	4.40E-01	6.01E+01
**CHILDREN**	Pb	6.17E-02	1.08E-01	2.19E+00	3.47E+00
	Cd	1.67E+00	1.17E+00	2.43E+01	5.94E+02
	Ni	3.89E-01	2.08E-02	1.32E+00	5.05E+00
	Cr	1.53E+00	1.53E+00	1.16E+02	4.17E+01
	Zn	3.94E-03	1.62E-03	3.10E-02	3.32E+00
	As	1.39E+00	1.39E+00	4.86E+00	4.85E+01
	Fe	3.95E-02	4.05E-02	6.42E-01	8.77E+01
**Hazard Index**	Adult	3.48E+00	2.92E+00	1.03E+02	5.37E+02
	Children	5.08E+00	4.25E+00	1.50E+02	7.84E+02

## 4. Conclusions

The occurrence of heavy metals in landfills leachate has been investigated in a major municipal area in Uyo, Nigeria. The elemental compositions of impacted surface water and sediment samples were determined. Analyses were conducted using standard analytical procedures and methods. The results indicated that municipal solid waste leachate, surface water, and sediment samples all contained elevated concentrations of heavy metals, implying a significant influence of seeping leachate from the dumpsites. Concentrations of heavy metals in the impacted freshwater ecosystem are season-dependent and variable. Pollution indicators revealed that the sediment samples examined were low to moderately polluted by toxic elements from the investigated non-sanitary landfills. Standard risk models were used to evaluate the significant threats posed by these toxic elements to human health. Elevated levels of these potentially toxic heavy metals in leachate from the non-sanitary landfills indicated a statistically significant carcinogenic lifetime risk to adults, children, and kids, owing to the landfill leachate’s ability to bioaccumulate and be distributed predominantly through the surrounding soils into the groundwater. In the case of adults, children, and kids, the incremental lifetime cancer rate (ILCR) values were within the tolerable range of 1.00E-06–1.00E-04. The lifetime carcinogenicity risks associated with oral ingestion exposure to heavy metals were 9.09E-05 for kids, but 1.21E-05 and 3.60 E-05 for adults and children, respectively. Furthermore, the mean cumulative risk values for dermal exposures were 3.24E-07, 1.89E-06, and 1.17E-05 for adults, children, and kids, respectively. The findings highlight the potential dangers of human and biota exposure to pollutants in MSW landfills and reaffirm the importance of limiting fish and water intake from the impacted ecosystems associated with the landfills. These findings reinforce the need for routine monitoring to ascertain the safety status of humans and resources close to the dumpsites. Such surveillance would provide useful insight into individual, population, and overall ecosystem quality.
