# Correction: Occurrence and spatial distribution of heavy metals in landfill leachates and impacted freshwater ecosystem: An environmental and human health threat

**DOI:** 10.1371/journal.pone.0304882

**Published:** 2024-05-31

**Authors:** Joseph P. Essien, Donald I. Ikpe, Edu J. Inam, Aniefiokmkpong O. Okon, Godwin A. Ebong, Nsikak U. Benson

The third author’s name is spelled incorrectly. The correct name is: Edu J. Inam. The correct citation is: Essien JP, Ikpe DI, Inam EJ, Okon AO, Ebong GA, et al. (2022) Occurrence and spatial distribution of heavy metals in landfill leachates and impacted freshwater ecosystem: An environmental and human health threat. PLOS ONE 17(2): e0263279. https://doi.org/10.1371/journal.pone.0263279.
